# Effects of Placebo Interventions on Subjective and Objective Markers of Appetite–A Randomized Controlled Trial

**DOI:** 10.3389/fpsyt.2018.00706

**Published:** 2018-12-18

**Authors:** Verena Hoffmann, Marina Lanz, Jennifer Mackert, Timo Müller, Matthias Tschöp, Karin Meissner

**Affiliations:** ^1^Institute of Medical Psychology, Faculty of Medicine, LMU Munich, Munich, Germany; ^2^Helmholtz Diabetes Center, Helmholtz Zentrum München, German Research Center for Environmental Health (GmbH), Munich, Germany; ^3^German Center for Diabetes Research (DZD), Garching bei München, Germany; ^4^Division of Health Promotion, Coburg University of Applied Sciences, Coburg, Germany

**Keywords:** placebo effect, expectation, appetite, satiety, ghrelin

## Abstract

**Objective:** Patients' expectations about the benefit of an intervention are important determinants of the placebo effect. Little is known about the extent to which expectations influence outcomes of treatments in the field of appetite regulation. This study aimed to investigate the effects of treatment-related expectations on subjective and objective markers of appetite.

**Methods:** 90 healthy participants of normal weight were randomly allocated to either an appetite-enhancing placebo group, a satiety-enhancing placebo group, or a control group. All participants received a placebo capsule along with group-specific verbal suggestions to either be appetite-promoting, or satiety-enhancing, or to have no effect on appetite. Before and during the 2 h following randomization, participants were repeatedly asked to rate feelings of hunger and satiety on visual analog scales (VAS), and blood samples were taken repeatedly to assess plasma ghrelin levels as a physiological marker of hunger.

**Results:** In comparison to the control group, the satiety-enhancing placebo intervention significantly reduced appetite and increased satiety. The appetite-enhancing placebo intervention did not alter subjective levels of hunger, but increased plasma ghrelin levels in females.

**Conclusions:** Results provide the first experimental evidence that appetite-regulating placebo interventions can elicit a psychobiological response. Expectations are important factors to consider when evaluating the effects of interventions in the field of appetite regulation.

## Introduction

Obesity is a dramatically increasing problem in our society. Treatment approaches for obesity include psychological, pharmacological, and surgical interventions (1–3). To what extent placebo effects, i.e. positive treatment expectations, contribute to the success of obesity treatments is unclear. A recent systematic review of placebo-controlled surgery trials revealed that patients receiving sham bariatric surgery showed on average 71% of the weight loss reported by the patients in the active surgery groups (4). These data suggest a strong inhibitory effect of placebo interventions on appetite.

Eating behavior is closely linked to mental sets. For example, Higgs (5) reported that participants consumed less in a test session when they were reminded of a recent meal. Furthermore, Provencher et al. (6) showed that participants ate less when the meal was perceived as healthy. Crum and colleagues went one step further and evaluated the impact of expectations on plasma levels of the gut hormone ghrelin, a physiological marker of appetite (7). In a within-subjects design, healthy volunteers on two occasions were made believe to receive either a “high-caloric, indulgent milk shake” or a “low-caloric, sensitive milk shake.” In truth, both milk shakes were of identical contents. Results showed a different ghrelin response to these milk shakes: In comparison to the “sensible” shake, the ghrelin increase was larger when expecting the “indulgent” milk shake, followed by a sharper decline of ghrelin levels 30 min after drinking the shake. These findings indicate a strong impact of nutrition-specific expectations on appetite and satiety, as evidenced by altered plasma ghrelin levels before and after a test meal. Additionally, differences in eating behavior are linked to gender. Several studies have shown that females tend to eat healthier than men [i.e., avoiding high-fat food and eating more fruit and fiber; (8, 9)]. This has been linked to more concerns of women about their body weight as compared to men (10). Also, it has been reported, that females eat more sweets when perceiving stress than men (11).

In this study, we investigated whether treatment-related expectations can affect appetite, satiety and plasma ghrelin levels. In a between-subjects design, normal-weight participants received a placebo capsule together with the information that its content would either increase appetite, or increase satiety, or would leave appetite and satiety unaffected. We hypothesized that the appetite-enhancing placebo intervention would decrease satiety and increase appetite and plasma ghrelin levels, while the satiety-enhancing placebo intervention would have the opposite effects, both in comparison to a no treatment control group.

## Materials and Methods

### Participants

The study was conducted at the Institute of Medical Psychology at the LMU Munich, Germany. Healthy participants aged 18–36 years were recruited via flyers and a university mailing list. All participants had to be of normal weight (Body Mass Index (BMI) 18–25 kg/m^2^). Exclusion criteria included report of pregnancy, breastfeeding, regular use of medication (except hormonal contraceptives and anti-allergic drugs), acute or chronic disease, smoking, surgery in the last 4 weeks before the experiment, elevated fasting blood glucose levels (>100 mg/dl), and elevated levels of anxiety and/or depression scores [>7 in at least one subscale of the Hospital Anxiety and Depression Scale (HADS); (12)]. The study protocol was approved by the ethics committee of the Medical Faculty at LMU Munich. Participants provided written informed consent and received 45 Euro compensation.

### Experimental Procedure

**Figure 1:**
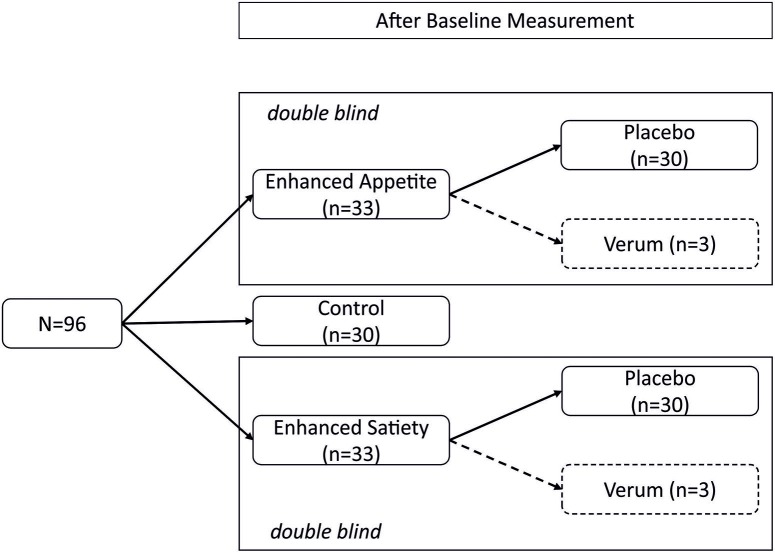
Study design. This randomized, double-blind, controlled trial was conducted in a between-subject design. After the baseline measurement, participants were randomly allocated to one of five groups (“control,” “placebo enhanced appetite,” “placebo enhanced satiety,” “verum enhanced appetite,” and “verum enhanced satiety”). The verum groups served only for double-blinding and were not evaluated further.

Ninty participants were randomly allocated to one of three groups: “control,” “enhanced appetite” (placebo), or “enhanced satiety” (placebo). To allow for double-blinding, 6 additional participants were randomized to verum treatments (3 enhanced appetite, 3 enhanced satiety; compare (13); Figure 1). Groups were stratified by sex due to sex differences in eating behavior (14) and neurobiological mechanisms of placebo effects (15). At recruitment, participants were informed that the experiment investigated how biological and psychological factors contribute to the regulation of hunger and appetite.

Participants underwent a single test session starting at 8 o'clock in the morning. They were asked to abstain from food for 10–12 h prior to the experiment (intake of small amounts of water was allowed). Upon arrival, participants took seat in a comfortable chair, and blood glucose levels were determined from finger blood samples using a BG Star device (Sanofi-Aventis, Hannover, Germany). An indwelling flexible catheter was then placed in the antecubital vein and kept patent with a saline infusion to allow for repeated blood drawing during the experiment. Electrodes to measure the electrocardiogram (ECG) were attached. Participants were then asked to fill in the “Hospital Anxiety and Depression Scale” [HADS; (12)], the ‘Three Factor Eating Questionnaire' [TFEQ; (16)] and to rate current levels of hunger and satiety on 100-mm visual analog scales (VAS). Thereafter, the first blood sample to assess ghrelin levels was collected and the ECG measurement was started. Following a 15-min baseline period, the experimenter opened the randomization envelope, performed the verbal expectancy manipulation according to group allocation (“appetite increase,” “satiety increase,” or “control”) and asked the participants to swallow the provided test capsule with 100 ml of mineral water (standardized temperature of 20°C). After resting periods of 30 and 60 min, respectively, participants were asked to rate current levels of hunger and satiety, and the second and third blood sample for ghrelin assessments were collected.

### Blinding and Randomization

A person not directly involved in the experiments prepared an opaque, sequentially-numbered randomization envelope for each participant according to a computer-generated randomization list. The envelopes contained information on the type of intervention (“appetite-stimulating,” “satiety-enhancing,” or “control”) as well as a test capsule. Neither the experimenter nor the participants were informed whether the capsule in the hunger-enhancing and satiety-enhancing conditions was a placebo or contained an active ingredient (double-blinded design).

### Capsules

Identical white and opaque vegetarian capsules were used for all interventions. The placebo capsules were filled with lactose (Heirler Cenovis GmbH, Radolfzell, Germany). For the satiety-increasing active intervention, capsules were filled with an alginate complex (lyophilized sodium-alginate complex, added with aluminum- and calciumchoride; CM3 Alginat Kapseln, Easyway GmbH, Monheim, Germany). Alginate reduces hunger and increases satiety feelings, which is partly due to its volume-increasing content (17). For the appetite-stimulating active intervention, one tablet of “Appetit-Anreger” with extracts of bitter herbs (Zirkulin Naturheilmittel GmbH, Bremen) was placed in the study capsules. Dietary supplements containing bitters are traditionally used to increase appetite and to support digestion (18).

### Expectancy Manipulation

Standardized expectancy manipulations were performed by two female experimenters in white coats (one undergraduate, one graduate student). After opening the randomization envelopes, participants in the appetite-stimulating groups were told to receive either a placebo capsule or a capsule filled with bitters, and that bitters are known to increase secretion of digestive fluids in the stomach and thus are expected to increase appetite within 20–30 min after intake of the capsule. Participants in the satiety-enhancing groups were told to receive either a placebo capsule or a capsule containing alginate, and that alginate is known to increase its volume in the stomach and thus is expected to enhance satiety within 20–30 min after intake of the capsule. They were told to receive either a verum or a placebo intervention (randomization ratio was not disclosed). Participants in the control group received a placebo capsule together with the information that its ingredients would have no effect on gastric activity and appetite.

### Measurements

#### Hunger and Satiety Ratings

Perceived hunger (“How hungry do you feel?”) was rated using a 100-mm visual analog scale from “0” (“not at all hungry”) to “100” (“extremely hungry”). Perceived satiety (“How full do you feel?”) were assessed by means of a 100-mm visual analog rating scale, ranging from 0 (“not at all full”) to 100 (“extremely full”).

#### Plasma Ghrelin

To assess the concentration of ghrelin in plasma, blood samples were collected in commercially available EDTA tubes (2.7 ml), complemented with 54 μl 4 mM 4-(2-aminoethyl)benzenesulfonyl fluoride hydrochloride (AEBSF) (19). Blood samples were immediately stored on ice and centrifuged within 30 min for 10 min at 3,000 g and 4°C. Two samples of 500 μl plasma were transferred to Eppendorf tubes and complemented with 100 μl 1 mM HCl. Samples were gently mixed and stored at −70°C until final analysis. Analysis of plasma ghrelin content was performed with the Ghrelin (total) Assay Kit (Catalogue number: EZGRT-89K) from Merck Millipore, Darmstadt, Germany according to protocol.

#### Electrocardiogram

The electrocardiogram was recorded to evaluate changes in heart rate. A transient increase of heart rate has been described as part of the cephalic phase response when food is anticipated (20). The electrocardiogram signal was measured continuously during the experiment using three disposable Ag/AgCl electrodes, which were positioned in an Einthoven Lead I configuration and connected to the BIOPAC amplifier module ECG100C of a BIOPAC MP 150 device (BIOPAC Systems Inc., Goleta, CA, USA). Data was acquired using AcqKnowledge 3.7.2 software and a sampling rate of 500 Hz. Intervals between successive R peaks (cardiac periods) were extracted from the electrocardiogram signal using the peak-detection function implemented in AcqKnowledge 3.7.2. Heart periods were examined and screened for artifacts based on the procedure developed by Proges and Byrne (21). Average heart rate was calculated for the last five artifact-free minutes of the baseline period and the two post-intervention periods (i.e., minutes 25–30 and minutes 55–60 after randomization).

#### Questionnaires

The Hospital Anxiety and Depression scale [HADS; (12)] was used to screen for anxiety and/or depression. The Three Factor Eating Questionnaire [TFEQ; (16)] with its three subscales “cognitive restraint of eating,” “disinhibition,” and “hunger” was used to test for possible differences in eating behavior between groups at baseline.

Female participants were asked for the normal length of their menstrual cycle, the beginning of the last menstruation, and whether they took hormonal contraceptives. Time point of ovulation was estimated by subtracting 14 days from the length of the menstrual cycle (22).

### Statistical Analyses

Assuming an effect size partial eta-squared of 0.1, the study was planned to have a power of 90% to detect a significant interaction effect between “group” and “time point” in a mixed ANOVA for changes of hunger, satiety and ghrelin from before to after the intervention (with a type 1 error of 5%) (calculated by GPower Version 3.1.7). However, we later decided to use ANCOVAs to adjust for the slight group differences at baseline. Statistical analyses were performed using SPSS (version 23.0). Hunger ratings, satiety ratings and plasma ghrelin levels were each subjected to 3-way analyses of covariance (ANCOVA), with two levels of “time” (30 min and 60 min after randomization), three levels of “group” (appetite, satiety, control) and two levels of “sex” (male, female). In each model, baseline levels (15 min before randomization) were included as covariates. Bonferroni corrections were applied, where appropriate. A *p*-value ≤ 0.05 (2-sided) was considered statistically significant.

## Results

### Participants

One Hundred thirteen participants were assessed for eligibility and 17 were excluded (three did not meet inclusion criteria, 12 declined to participate, one did not show up and one had elevated fasting blood glucose levels). Thirty participants each were assigned to the appetite group, the satiety group, and the control group. All participants completed the experiment.

Study groups were comparable with respect to demographic variables, eating behavior as well as anxiety and depression scores (Table 1). Participants had a mean age of 26.6 years (3.2 SD; range: 18–36 years) and a mean BMI of 21.9 kg/m^2^ (1.8 SD; range: 18.6–25 kg/m^2^). Fourteen women were in the preovulatory phase and two women in the postovulatory phase of the menstrual cycle, while 29 women were using hormonal contraceptives.

**Table 1:** Group characteristics at baseline.

**Variable**	**Appetite group (*n* = 30) mean (*SD*)**	**Satiety group (*n* = 30) mean (*SD*)**	**Control group (*n* = 30) mean (*SD*)**	***F* (*df*)**	***p*-value**
Age (years)	23.6 (3)	23.4 (2.9)	24 (3.7)	0.243 (2.87)	0.785
Body Mass Index (kg m^2^)	21.8 (2)	21.7 (1.7)	22.2 (1.7)	0.632 (2.87)	0.534
Blood Glucose (mg/dl)	93.8 (8.1)	95.3 (9.9)	95.5 (6.6)	0.337 (2.84)	0.715
Hunger ratings (VAS)	4.6 (3.2)	6.3 (2.3)	5.7 (2.6)	2.867 (2.87)	0.062
Satiety ratings (VAS)	3.0 (2.2)	2.6 (2.4)	2.6 (1.9)	1.931 (2.87)	0.151
Ghrelin levels (pg/ml)	495.2 (217.3)	463.2 (180.5)	464 (212.4)	0.215 (2.87)	0.807
**HOSPITAL ANXIETY AND DEPRESSION SCALE (HADS)**					
Anxiety	3 (2.4)	2.8 (1.9)	3.4 (1.6)	0.560 (2.87)	0.573
Depression	1.4 (1. 5)	1.6 (1.8)	1.9 (2.1)	0.652 (2.87)	0.523
**THREE FACTOR EATING QUESTIONNAIRE (TFEQ)**					
Cognitive Restraint of Eating	10.6 (2)	10.9 (2.2)	10.1 (2.0)	0.317 (2.87)	0.317
Disinhibition	7.7 (2)	8.3 (2.1)	8.1 (2.2)	0.624 (2.87)	0.538
Hunger	6.3 (2.2)	6.8 (2)	7 (1.9)	0.957 (2.87)	0.388

*SD, Standard Deviation*.

### Hunger Ratings

**Figure 2:**
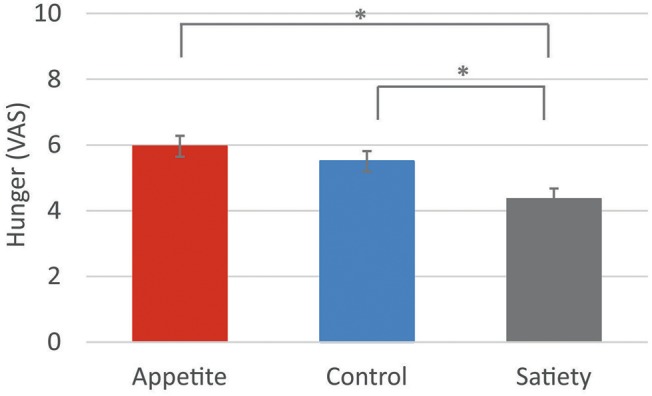
Hunger ratings. Baseline-corrected hunger ratings following the three placebo interventions (estimated means ± SEM). VAS = visual analogue scale. *p < 0.05.

The 3-way ANCOVA for post-intervention hunger ratings, controlled for baseline levels, revealed a significant 3-way interaction between “group,” “time,” and “sex” [*F*_group × *time*×*sex*(2, 83)_ = 4.0, *p* = 0.023]. However, *post hoc* 2-way ANCOVAs performed separately for each sex showed no significant interaction effect between “group” and “time” [women, *F*_group × *time*(2, 41)_ = 3.1, *p* = 0.058; men, *F*_group × *time*(2, 41)_ = 0.6, *p* = 0.571). Furthermore, the 3-way ANCOVA showed a significant main effect of “group” [*F*_group_ (2, 83) = 6.7, *p* = 0.002]. Bonferroni-corrected *post hoc* tests indicated significantly lower hunger ratings in the satiety group compared to the control group (*p* = 0.033) and to the appetite group (*p* = 0.002) (Figure 2, Table 2).

**Table 2:** Post-intervention values (baseline-adjusted) of hunger ratings, satiety ratings, plasma ghrelin levels, and heart rate.

	**Appetite group**		**Satiety group**		**Control group**	
	**Males (*n* = 15) Mean (SE)**	**Females (*n* = 15) Mean (SE)**	**Males (*n* = 15) Mean (SE)**	**Females (*n* = 15) Mean (SE)**	**Males (*n* = 15) Mean (SE)**	**Females (*n* = 15) Mean (SE)**
Hunger ratings (VAS; cm)	6.3 (0.4)	5.5 (0.5)	3.8 (0.4)	5.0 (0.5)	5.8 (0.4)	5.2 (0.5)
Satiety ratings (VAS; cm)	2.5 (0.4)	2.1 (0.4)	4.0 (0.4)	3.9 (0.4)	2.3 (0.4)	2.3 (0.4)
Plasma ghrelin levels (pg/ml)	453.5 (20.2)	535.5 (20.1)	455.6 (20.8)	478.9 (21.0)	504.1 (24.2)	452.8 (20.1)
Heart rate (1/min)	63.2 (1.7)	63.3 (1.7)	65.7 (1.7)	62.2 (1.7)	62.8 (1.7)	64.6 (1.7)

*SE, Standard Error; VAS, Visual Analogue Scale*.

### Satiety Ratings

**Figure 3:**
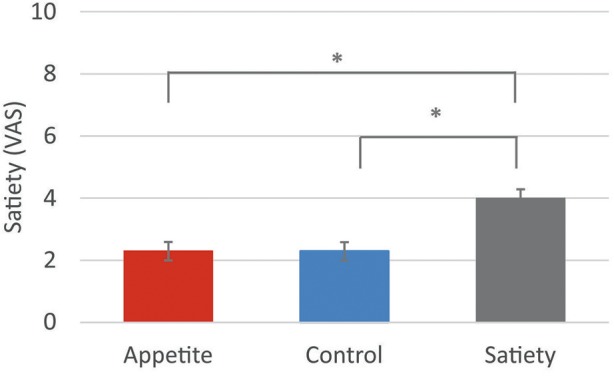
Satiety ratings. Baseline-corrected satiety ratings following the three placebo interventions (estimated means ± SEM). VAS = visual analogue scale. *p < 0.05.

The 3-way ANCOVA for post-intervention satiety ratings, controlled for baseline levels, revealed a significant main effect of “group” [*F*_(2, 83)_ = 11.1, *p* < 0.001]. Bonferroni-corrected *post hoc* tests indicated significantly higher satiety ratings in the satiety group than in the control group (*p* < 0.001) and in the appetite group (*p* < 0.001) (Figure 3, Table 2). The 3-way interaction between “group,” “time,” and “sex” was not significant [*F*_group × *time*×*sex*(2, 83)_ = 2.5, *p* = 0.102].

### Ghrelin Levels

**Figure 4:**
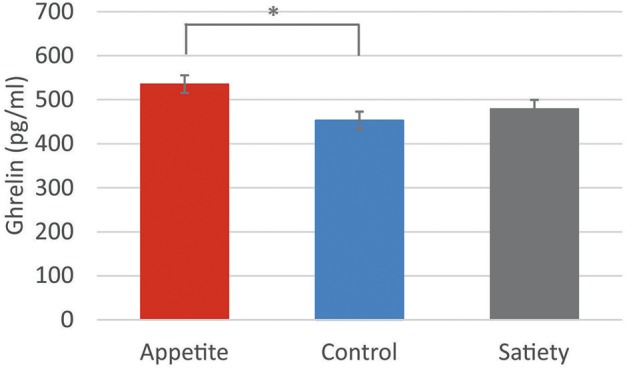
Ghrelin levels in women. Baseline-corrected plasma ghrelin levels (pg/ml) in women following the three placebo interventions (estimated means ± SEM). *p < 0.05.

The 3-way ANCOVA for post-intervention plasma ghrelin levels, controlled for baseline levels, revealed a significant 2-way interaction between “group” and “sex” [*F*_(2, 71)_ = 3.4, *p* = 0.040]. Separate ANCOVAs for male and female participants indicated a significant main effect of “group” in women [*F*_(2, 37)_ = 4.4, *p* = 0.019] but not in men [*F*_(2, 33)_ = 1.5, *p* = 0.235]. Bonferroni-corrected *post hoc* tests indicated that the interaction in women was due to higher post-intervention ghrelin levels in the appetite group compared to the control group (*p* = 0.019; Figure 4, Table 2). Neither the main effect of “group” [*F*_group(2, 71)_ = 0.9, *p* = 0.401) nor the 3-way interaction between “group,” “time,” and “sex” [F_group × *time*×*sex*(2, 71)_ = 2.7, *p* = 0.075] was significant.

### Heart Rate

The 3-way ANCOVA for post-intervention heart rate, controlled for baseline levels, revealed no significant main or interaction effects [*F*_group(2, 82)_ = 0.1, *p* = 0.922; *F*_group × *time*(2, 82)_ = 0.2, *p* = 0.812; *F*_group × *time*×*sex*(2, 82)_ = 1.1, *p* = 0.342; Table 2].

### Treatment Guesses

Thirteen participants (72.2%) in the satiety group, but only five participants (27.8%) in the appetite group guessed to having received the active agent. The difference between groups was significant (χ2 = 0.1, *p* = 0.024).

## Discussion

This is the first study designed to evaluate the effects of treatment-related expectations on appetite, satiety, and associated plasma ghrelin levels. Our randomized-controlled double-blinded experiment revealed that the satiety-enhancing placebo intervention successfully altered subjective feelings of appetite and satiety in the suggested direction. Furthermore, the appetite-enhancing placebo intervention increased ghrelin levels in women.

A recent meta-analysis of sham-controlled surgery trials suggested that bariatric surgery for obesity is associated with a large placebo effect on weight loss, equaling 71% of the effect of active bariatric surgery (4). Our finding that the satiety-enhancing placebo intervention indeed increased satiety provides the first experimental evidence that treatment-related expectations contribute to the success of satiety-enhancing medical interventions.

The retrospective evaluation of treatment guesses suggests that the appetite-enhancing placebo intervention was less credible to the participants than the satiety-enhancing placebo intervention. This could explain why the appetite-enhancing placebo intervention did not alter subjective feelings of appetite and satiety. However, the guess of having received placebo does not necessarily mean that the participant did not believe in the effectiveness of the intervention. Recent studies indicate that also open-label placebo administration can lead to positive beliefs and symptom improvement (23–25). Supporting this explanation, we observed an increase in ghrelin levels following the appetite-enhancing placebo intervention in women, suggesting the occurrence of a placebo effect on a physiological level. Ghrelin is secreted by the stomach, with levels peaking just before a meal and declining after feeding. In addition, ghrelin serves as an interoceptive signal for food-seeking behavior (26, 27). A previous study in a predominantly female cohort (65% women) found that plasma ghrelin levels increased when participants anticipated the intake of an “indulgent” milk-shake as compared to a “low-calorie” milk-shake, even though hunger ratings did not change (7). This may indicate that ghrelin is a highly sensitive measure to capture a psychologically mediated increase in appetite that occurs even before behavioral effects are measureable. With regard to the observed sex difference, it is important to note that stronger physiological placebo responses in women have also been reported in studies of placebo analgesia (15, 28). In addition, there is ample evidence that the physiology of appetite differs between sexes (29, 30). For example, women showed higher brain activation in the fusiform gyrus while viewing high-caloric pictures in the hungry state (31). Furthermore, brain activation to calorie-rich foods within the dorsolateral, ventrolateral, and ventromedial prefrontal cortices, the middle/posterior cingulate, and the insula were larger in women than in men (32), regions that play a role in self-reflection (33). Interestingly, sex differences in eating behavior are mediated, among other factors, by the gut hormone ghrelin (14), both in terms of secretion of this hormone and of ghrelin sensitivity (34, 35). Thus, the sex-specific ghrelin response in our experiment is in line with previous studies showing a stronger physiological response to placebo interventions as well as to appetite-enhancing food stimuli in women.

Our results provide first evidence that a placebo intervention to enhance appetite may enhance ghrelin secretion in women even before behavioral effects are measureable. In contrast, we found a strong effect of the satiety-enhancing intervention on ratings of hunger and satiety, notably without changes in circulating levels of total ghrelin. These data collectively suggest that ghrelin secretion is most likely unrelated to the placebo effect on satiety. It could be argued that food ingestion is a prerequisite for the postprandial fall in circulating ghrelin. However, as demonstrated in healthy human volunteers, postprandial suppression of ghrelin secretion did not differ between subjects receiving a mixed meal or who have been sham fed to allow smelling, chewing and tasting but not swallowing of food (26, 36). An anorexigenic hormone such as leptin or peptide YY (37) may still be better suited to capture the hormonal correlates of the placebo effect on satiety.

Several limitations of our results need to be mentioned. First, the short observational period in our experiment does not allow any conclusion on whether placebo effects on hunger and satiety can last longer than a few hours. Second, we performed our experiment in a normal-weight sample. Further studies are needed to clarify whether the findings of our experiment can be replicated in obese and anorectic patients. Third, our study was designed to investigate placebo effects on hunger and satiety induced by verbal suggestions. Learning mechanisms, such as behavioral conditioning and reinforcement learning, are known to affect eating behavior (38) as well as placebo effects (39), and their involvement in placebo effects on appetite-regulation should be evaluated in follow-up studies.

In conclusion, the results of the present study indicate a powerful inhibition of appetite in response to a satiety-enhancing placebo intervention and first evidence for an increase of ghrelin levels in women in response to an appetite-enhancing placebo intervention. Results thus provide the first experimental evidence that expectations are important factors to consider when evaluating the effects of medical interventions in the field of appetite regulation. Further studies with additional physiological outcome parameters are needed to better understand the psychobiological processes triggered by appetite-modulating placebo interventions.

## Author Contributions

VH and KM designed the experiments. VH, ML and JM performed the experiments. VH and KM analyzed the data. VH, TM, MT, and KM interpreted the data. VH drafted the first version of the manuscript. All authors critically reviewed the manuscript.

### Conflict of Interest Statement

The authors declare that the research was conducted in the absence of any commercial or financial relationships that could be construed as a potential conflict of interest.
